# Distinguishing Low Expression Levels of Human Epidermal Growth Factor Receptor 2 in Breast Cancer: Insights from Qualitative and Quantitative Magnetic Resonance Imaging Analysis

**DOI:** 10.3390/tomography11030031

**Published:** 2025-03-10

**Authors:** Yiyuan Shen, Xu Zhang, Jinlong Zheng, Simin Wang, Jie Ding, Shiyun Sun, Qianming Bai, Caixia Fu, Junlong Wang, Jing Gong, Chao You, Yajia Gu

**Affiliations:** 1 Department of Radiology, Fudan University Shanghai Cancer Center, 270 Dongan Road, Shanghai 200032, China23211320011@m.fudan.edu.cn (J.Z.);; 2 Department of Oncology, Shanghai Medical College, Fudan University, 270 Dongan Road, Shanghai 200032, China; baiqianming@163.com; 3 Department of Pathology, Fudan University Shanghai Cancer Center, 270 Dongan Road, Shanghai 200032, China; 4 MR Application Development, Siemens Shenzhen Magnetic Resonance Ltd., Shenzhen 518057, China; 5 Department of Nuclear Medicine, Insel Hospital, University of Bern, 3010 Bern, Switzerland; junlong.wang@students.unibe.ch

**Keywords:** breast cancer, human epidermal growth factor receptor 2, magnetic resonance imaging, low HER2 expression

## Abstract

Background: The discovery of novel antibody–drug conjugates for low-expression human epidermal growth factor receptor 2 (HER2-low) breast cancer highlights the inadequacy of the conventional binary classification of HER2 status as either negative or positive. Identification of HER2-low breast cancer is crucial for selecting patients who may benefit from targeted therapies. This study aims to determine whether qualitative and quantitative magnetic resonance imaging (MRI) features can effectively reflect low-HER2-expression breast cancer. Methods: Pre-treatment breast MRI images from 232 patients with pathologically confirmed breast cancer were retrospectively analyzed. Both clinicopathologic and MRI features were recorded. Qualitative MRI features included Breast Imaging Reporting and Data System (BI-RADS) descriptors from dynamic contrast-enhanced MRI (DCE-MRI), as well as intratumoral T2 hyperintensity and peritumoral edema observed in T2-weighted imaging (T2WI). Quantitative features were derived from diffusion kurtosis imaging (DKI) using multiple b-values and included statistics such as mean, median, 5th and 95th percentiles, skewness, kurtosis, and entropy from apparent diffusion coefficient (ADC), D_app_, and K_app_ histograms. Differences in clinicopathologic, qualitative, and quantitative MRI features were compared across groups, with multivariable logistic regression used to identify significant independent predictors of HER2-low breast cancer. The discriminative power of MRI features was assessed using receiver operating characteristic (ROC) curves. Results: HER2 status was categorized as HER2-zero (n = 60), HER2-low (n = 91), and HER2-overexpressed (n = 81). Clinically, estrogen receptor (ER), progesterone receptor (PR), hormone receptor (HR), and Ki-67 levels significantly differed between the HER2-low group and others (all *p* < 0.001). In MRI analyses, intratumoral T2 hyperintensity was more prevalent in HER2-low cases (*p* = 0.009, *p* = 0.008). Mass lesions were more common in the HER2-zero group than in the HER2-low group (*p* = 0.038), and mass shape (*p* < 0.001) and margin (*p* < 0.001) significantly varied between the HER2 groups, with mass shape emerging as an independent predictive factor (HER2-low vs. HER2-zero: *p* = 0.010, HER2-low vs. HER2-over: *p* = 0.012). Qualitative MRI features demonstrated an area under the curve (AUC) of 0.763 (95% confidence interval [CI]: 0.667–0.859) for distinguishing HER2-low from HER2-zero status. Quantitative features showed distinct differences between HER2-low and HER2-overexpression groups, particularly in non-mass enhancement (NME) lesions. Combined variables achieved the highest predictive accuracy for HER2-low status, with an AUC of 0.802 (95% CI: 0.701–0.903). Conclusions: Qualitative and quantitative MRI features offer valuable insights into low-HER2-expression breast cancer. While qualitative features are more effective for mass lesions, quantitative features are more suitable for NME lesions. These findings provide a more accessible and cost-effective approach to noninvasively identifying patients who may benefit from targeted therapy.

## 1. Introduction

Breast cancer is one of the most common malignancies affecting women worldwide [1]. A key therapeutic target and prognostic marker in breast cancer is the human epidermal growth factor receptor 2 (HER2) [2,3]. Traditionally, HER2 status is classified as either positive or negative based on immunohistochemistry (IHC) and fluorescence in situ hybridization (FISH) testing. HER2-positive patients benefit from targeted therapies [3], while HER2-negative patients lack such options [4,5]. However, the development of novel antibody–drug conjugates has introduced a new treatment pathway for HER2-low breast cancers, a subset of HER2-negative patients constituting around 50% of the breast cancer population, which improves the overall survival of HER2-low patients [6]. Furthermore, low HER2 expression has distinct biological behaviors and clinical outcomes compared with zero HER2 expression, highlighting the limitations of the conventional binary classification [7,8]. This underscores the need to redefine HER2 status into three categories: HER2-zero, HER2-low, and HER2-overexpression.

Magnetic resonance imaging (MRI) has proven valuable for its noninvasive, high-resolution capabilities in differentiating HER2-positive from HER2-negative breast cancer [9,10,11]. For example, ELIAS et al. demonstrated a correlation between HER2-positive breast cancer and rapid kinetics on dynamic contrast-enhanced MRI (DCE-MRI) [12]. Similarly, Li et al. developed a machine learning model based on DCE-MRI to predict HER2-positive status, achieving an area under the receiver operator characteristic (ROC) curve (AUC) of 0.713 [13]. However, the rising need to identify HER2-low status poses a new diagnostic challenge for MRI.

Few studies have focused on the imaging characteristics of HER2-low breast cancers, and most rely on advanced techniques like radiomics and deep learning [14,15,16]. For example, Bian et al. developed a multiparametric MRI radiomics model that differentiated HER2-low from HER2-negative breast cancers [14], while Guo et al. used deep learning to predict HER2 status and patient prognosis [15]. Despite their promise, these models are complex and have limited clinical applications due to their interpretative challenges. A simpler, more practical imaging method is needed to accurately reflect HER2 expression levels.

Our previous studies and other studies have found value in both qualitative and quantitative MRI analyses for the differential diagnosis of breast cancer [17,18,19]. Qualitative analysis provides anatomical insights into tumors and surrounding tissue morphology, while quantitative histogram features, particularly from diffusion techniques, are straightforward to obtain and interpret. Our previous study found background parenchymal enhancement to be valuable in the differential diagnosis of molecular subtypes of breast cancer, especially triple-negative breast cancer [20]. Moreover, our research showed that diffusion-based histogram parameters based on multi-b-value diffusion imaging could distinguish HER2 statuses [21].

This study aims to explore the potential of both qualitative and quantitative MRI features in reflecting low-HER2-expression breast cancer, particularly in the context of mass and non-mass enhancement (NME) lesions. We investigate the utility of Breast Imaging Reporting and Data System (BI-RADS) descriptors, intratumoral T2 hyperintensity, and peritumoral edema in qualitative analyses and diffusion kurtosis imaging (DKI) histogram metrics in quantitative analysis.

## 2. Materials and Methods

### 2.1. Patients

**Figure 1:**
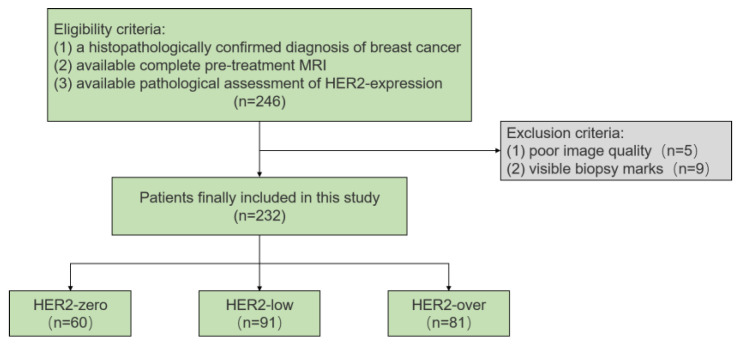
Flowchart of the study.

This retrospective study included 246 consecutive patients treated at the Fudan University Shanghai Cancer Center between January 2018 and December 2019. The inclusion criteria were as follows: (1) histopathologically confirmed breast cancer based on surgical specimens; (2) availability of complete pre-treatment MRI scans, including T1-weighted imaging (T1WI), T2-weighted imaging (T2WI), dynamic contrast-enhanced MRI (DCE-MRI), and multi-b-value DKI sequences, all performed within one month before surgery; and (3) available pathological assessment of HER2 expression. To ensure the accuracy of imaging assessments, fourteen patients were excluded due to poor-quality MRI images with severe motion and breathing artifacts or visible biopsy marks. Ultimately, 232 patients were included in the study. A flowchart of the patient selection process is shown in Figure 1.

The study protocol adhered to the Declaration of Helsinki and was approved by the ethical review committee of the Fudan University Shanghai Cancer Center, with a waiver for informed consent (NCT04461990).

### 2.2. Histopathological Analysis

Histopathological analysis was performed on surgical specimens. HER2 expression was determined using IHC scores, evaluated by expert pathologists following the 2018 guidelines from the American Society of Clinical Oncology and the College of American Pathologists [22]. Tumors with an IHC score of 2+ were further assessed using FISH. Based on the results, HER2 expression was classified into three categories: HER2-zero (IHC score of 0), HER2-low (IHC score of 1+ or 2+ with a negative FISH result), and HER2-overexpression (IHC score of 3+ or 2+ with a positive FISH result). Estrogen receptor (ER) and progesterone receptor (PR) positivity were defined using a cutoff value of 1%, and Ki-67 expression was categorized into high and low groups based on the median value.

### 2.3. Imaging Acquisition

Breast MRI examinations were performed using a 3T MRI scanner (MAGNETOM Skyra; Siemens Healthcare, Erlangen, Germany) with a dedicated 16-channel phased array bilateral breast coil. Patients were scanned in the prone position, and the sequences included axial T1WI, axial fast spin–echo fat-suppressed T2WI, axial DCE-MRI, and axial multi-b-value DKI. DCE-MRI comprised one pre-contrast phase and five post-contrast phases, while DKI was performed before contrast injection using four b-values (0, 800, 1400, and 2000 s/mm^2^). Detailed parameters for each sequence are provided in Table S1.

### 2.4. Imaging Analysis

#### 2.4.1. Qualitative MRI Analysis

Two experienced breast radiologists (Y.Y.S., with 5 years of experience, and C.Y., with 15 years of experience) independently reviewed all MRI data, blinded to the clinical and histopathological information. In cases of disagreement, a third radiologist (Y.J.G., with 30 years of experience in breast cancer imaging) made the final decision.

The following qualitative tumor features were recorded based on the American College of Radiology BI-RADS [23]: (1) fibroglandular tissue (FGT); (2) background parenchymal enhancement (BPE); (3) multifocal or multicentric lesions; (4) intratumoral T2 hyperintensity; (5) peritumoral edema; (6) lesion type; (7) lesion size; (8) shape, margin, and internal enhancement pattern of mass lesions; and (9) distribution and internal enhancement pattern of NME lesions. For multifocal or multicentric lesions, the largest lesion was analyzed.

#### 2.4.2. Quantitative MRI Analysis

Quantitative analysis included conventional apparent diffusion coefficient (ADC) values derived from the mono-exponential model, as well as $D_{app}$ and $K_{app}$ values from the DKI model. All DKI images were processed using a workstation (Body Diffusion Toolbox, version 0.2.2, Siemens Healthcare).

DKI is an extension of the commonly used diffusion weighted imaging (DWI) sequence in clinical practice. Traditionally, an ADC map is derived from the DWI sequence using the mono-exponential model. In this study, ADC, $D_{app}$**,** and $K_{app}$ were derived from the DKI sequence. ADC was calculated using the conventional b-values (0 and 800 s/mm^2^) based on the mono-exponential model, which is commonly used in clinical practice. $D_{app}$ and $K_{app}$ were calculated using all b-values (0, 800, 1400, 2000 s/mm^2^) based on the extended fitting model. The specific equations used are as follows:

For the ADC:

$$S\left(b\right)=S_{0}exp[-b\cdot ADC]$$

where $S\left(b\right)$ is the signal intensity at a given b value and $S_{0}$ is the signal intensity at b = 0.

For $D_{app}$ and $K_{app}$, all b-values (0, 800, 1400, 2000) were fitted using the following equation:

$$S\left(b\right)=S_{0}exp[-b\cdot D_{app}+\frac{1}{6}b^{2}\cdot {D_{app}}^{2}\cdot K_{app}]$$

where $S\left(b\right)$ is the signal intensity at a specified b value, $S_{0}$ is the signal intensity at b = 0. $K_{app}$ is the apparent kurtosis coefficient, which is a unitless parameter that indicates the deviation of the water motion from the Gaussian distribution. $D_{app}$ is similar to the ADC, adjusted for the Gaussian diffusion behavior of water molecules.

For each case, two radiologists manually delineated the volume of interest (VOI) layer by layer around the outer edge of the lesion on the ADC maps, avoiding areas of hemorrhage, necrosis, large vessels, and cysts. Both radiologists were trained and standardized in the VOI delineation process. These VOIs were then applied to the $D_{app}$ and $K_{app}$ maps. Quantitative tumor features obtained from the VOIs included the mean, median, 5th and 95th percentiles, skewness, kurtosis, and entropy. To ensure consistency in manual VOI delineation, an inter-observer reliability analysis was conducted using the intraclass correlation coefficient (ICC).

### 2.5. Statistical Analysis

Statistical analyses were conducted using SPSS 20.0 software. Categorical data were presented as proportions and analyzed using the chi-squared test or Fisher’s exact test. Continuous data were presented as medians with quartiles or means with standard deviations, depending on the normality of the distribution, and analyzed using the Mann–Whitney U test or Kruskal–Wallis test. All statistical tests were two-sided, with *p*-values < 0.05 considered statistically significant. Variables with *p*-values < 0.1 in univariate analysis were included in multivariate logistic regression analysis. Diagnostic performance was evaluated using receiver operating characteristic (ROC) analysis, and the area under the curve (AUC), along with sensitivity and specificity, was calculated. For inter-observer variability, categorical data were determined by kappa coefficients and continuous data by ICC, respectively. Kappa and ICC values were interpreted as follows: <0.2 (slight agreement), 0.21–0.40 (fair agreement), 0.41–0.60 (moderate agreement), 0.61–0.80 (good agreement), and 0.81–0.99 (excellent agreement).

## 3. Results

### 3.1. Clinicopathologic Characteristics

Among the 232 patients included in the study, 60 (25.9%) had HER2-zero breast cancer, 91 (39.2%) had HER2-low breast cancer, and 81 (34.9%) had HER2-overexpression breast cancer. Of these, 145 patients presented with solitary lesions, while 87 had multifocal or multicentric lesions. The majority of the cancers were invasive ductal carcinoma (230 cases), with one case of invasive lobular carcinoma and one case of metaplastic carcinoma.

Regarding clinicopathologic features, the HER2-low group showed significantly higher positive rates for ER, PR, and hormone receptor (HR) status than the HER2-zero and HER2-overexpression groups. Additionally, based on the median Ki-67 level of 40%, a higher proportion of HER2-low cases had low Ki-67 expression compared with the other groups. There were no significant differences in lesion size among the groups. Table 1 summarizes the clinicopathological features of all patients.

### 3.2. Qualitative MRI Features According to HER2 Expression Levels

Significant differences were observed in the intratumoral T2 hyperintensity and lesion types between HER2-low expression and the other groups. Intratumoral T2 hyperintensity was more frequently observed in HER2-low breast cancer than in HER2-zero and HER2-overexpressing cancers (*p* = 0.009 and *p* = 0.008, respectively). Additionally, mass lesions were more common in the HER2-zero group, while NME and mass with NME lesions were more prevalent in the HER2-overexpression group (Table 2).

To further explore these findings, lesion types were categorized based on the presence of NME into mass or NME-related lesions. For mass lesions, HER2-low breast cancers were more likely to have irregular shapes than the other two groups (*p <* 0.001 and *p* = 0.009, respectively). HER2-low breast cancers also more frequently showed non-circumscribed margins than HER2-zero cancers (*p <* 0.001). However, no significant differences were found in the qualitative MRI features for NME-related lesions (Table 2).

**Figure 2:**
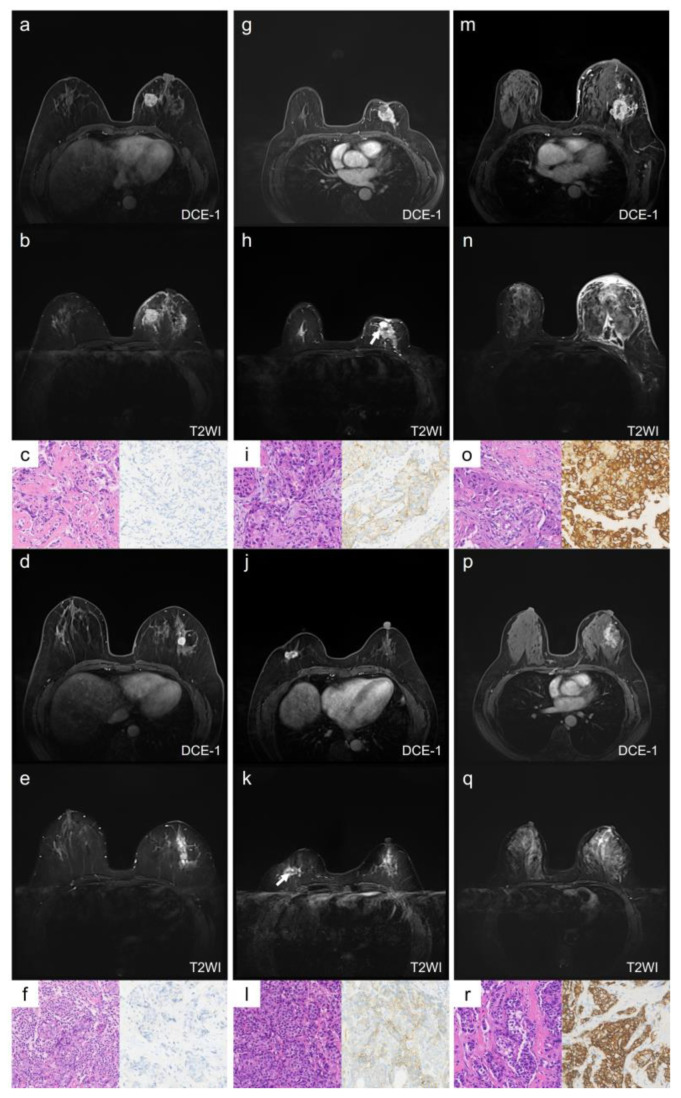
Representative images of qualitative MRI features in HER2-zero, HER2-low, and HER2-overexpression breast cancer. Case 1 (**a**–**c**) and Case 2 (**d**–**f**) show HER2-zero breast cancer, Case 3 (**g**–**i**) and Case 4 (**j**–**l**) show HER2-low breast cancer, and Case 5 (**m**–**o**) and Case 6 (**p**–**r**) show HER2-overexpression breast cancer. Case 1 (**a**–**c**): A 59-year-old female with HER2-zero expression breast cancer. The lesion is located in the left breast, has a rounded shape with circumscribed margins, and shows no NME (**a**). There is no intratumoral hyperintensity on T2WI (**b**). The HE staining image (×20, (**c**) left) shows invasive ductal carcinoma, and the IHC result (×20, (**c**) right) shows an HER2 score of 0. Case 2 (**d**–**f**): A 51-year-old female with HER2-zero expression breast cancer. The lesion is located in the left breast, has a rounded shape with circumscribed margins, and shows no NME (**d**). There is no intratumoral hyperintensity on T2WI (**e**). The HE staining image (×20, (**f**) left) shows invasive ductal carcinoma, and the IHC result (×20, (**f**) right) shows an HER2 score of 0. Case 3 (**g**–**i**): A 67-year-old female with low-HER2-expression breast cancer. The lesion is located in the left breast, has an irregular shape and uncircumscribed margins, and shows no NME (**g**). There is an intratumoral hyperintensity area (arrow) on T2WI (**h**). The HE staining image (×20, (**i**) left) shows invasive ductal carcinoma, and the IHC result (×20, (**i**) right) shows an HER2 score of 1+. Case 4 (**j**–**l**): A 59-year-old female with low-HER2-expression breast cancer. The lesion is located in the right breast, has an irregular shape and uncircumscribed margins, and shows no NME (**j**). There is an intratumoral hyperintensity area (arrow) on T2WI (**k**). The HE staining image (×20, (**l**) left) shows invasive ductal carcinoma, and the IHC result (×20, (**l**) left right) shows an HER2 score of 1+. Case 5 (**m**–**o**): A 61-year-old female with HER2-overexpression breast cancer. The lesion is located in the left breast, has an irregular shape and uncircumscribed margins, and shows NME (**m**). There is no intratumoral hyperintensity on T2WI (**n**). The HE staining image (×20, (**o**) left) shows invasive ductal carcinoma, and the IHC result (×20, (**o**) right) shows an HER2 score of 3+. Case 6 (**p**–**r**): A 55-year-old female with HER2-overexpression breast cancer. The lesion is located in the left breast, and shows as an NME lesion (**p**). There is no intratumoral hyperintensity on T2WI (**q**). The HE staining image (×20, (**r**) left) shows invasive ductal carcinoma, and the IHC result (×20, (**r**) right) shows an HER2 score of 3+. NME: non-mass enhancement; DCE-1: enhancement during the first phase.

In multivariate analysis, the qualitative MRI features with a *p*-value *<* 0.1 from the univariate analysis—intratumoral T2 hyperintensity, mass shape, and mass margin—were included (Table S2). The results showed that irregular shape remained an independent predictor of HER2-low breast cancer when compared with HER2-zero (odds ratio [OR] = 3.91, 95% confidence interval [CI]: 1.38–11.05, *p* = 0.010) and HER2-overexpression (OR = 2.23, 95% CI: 1.29–8.10, *p* = 0.012). The AUC for distinguishing HER2-low from HER2-zero was 0.763 (95% CI: 0.667–0.859), and for HER2-low from HER2-overexpression, the AUC was 0.663 (95% CI: 0.550–0.775). Representative cases of the qualitative MRI features for HER2-zero, HER2-low, and HER2-overexpression are illustrated in Figure 2.

### 3.3. Quantitative Diffusion Features by HER2 Expression Levels

The histogram analysis of quantitative diffusion parameters revealed significant differences between the HER2-low and HER2-overexpression groups (Table 3). Specifically, the HER2-low group had lower ADC_mean_, ADC_median_, ADC_5%_, D_mean_, D_median_, and D_5%_ values than the HER2-overexpression group (all *p* < 0.05). Conversely, the HER2-low group had significantly higher K_mean_, K_median_, K_95%_, and K_entropy_ values (all *p <* 0.05).

**Figure 3:**
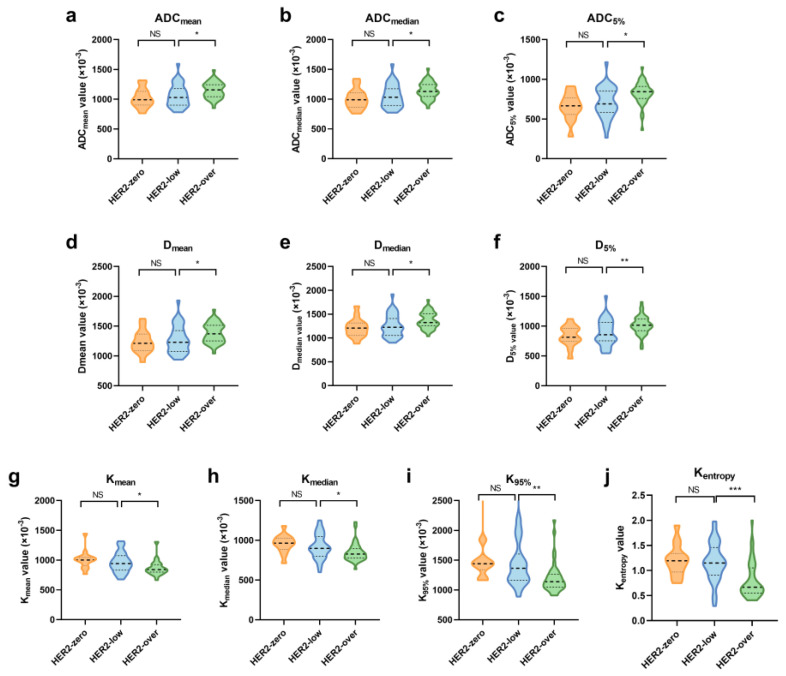
Violin graph of significant quantitative MRI features in NME-related lesions among the HER2-zero, -low, and -overexpression groups. (**a**–**c**) Histogram features of ADC; (**d**–**f**) histogram features of D_app_; (**g**–**j**) histogram features of K_app_. Significance levels: *: *p* < 0.05, **: *p* < 0.01, ***: *p* < 0.001; NS: not significant.

Further analysis of these quantitative features was conducted separately for mass and NME-related lesions. Significant differences in the diffusion parameters between HER2-low and HER2-overexpressing groups persisted for NME-related lesions (Figure 3, Table S3), but no significant differences were observed for mass lesions (Table S4).

ROC analysis was performed to assess the diagnostic performance of quantitative diffusion features in distinguishing HER2-low from HER2-overexpressing cancers for NME-related lesions (Table 4). The AUC values for individual histogram parameters ranged from 0.666 to 0.786. When combining variables (Combined_ADC_, Combined_D_, Combined_K_, Combined_All_), the AUC values ranged from 0.681 to 0.802, with the Combined_All_ model showing the best predictive performance (AUC = 0.802). The Delong test revealed that the AUC for Combined_All_ was significantly higher than that for Combined_ADC_ (*p* = 0.029) and Combined_D_ (*p* = 0.003) but not significantly different from that for Combined_K_ (*p* = 0.54).

### 3.4. Reliability of Qualitative and Quantitative Features Analysis

The kappa coefficients for qualitative MRI features ranged from 0.820 to 0.957, indicating excellent agreement. The ICCs for ADC, D_app_, and K_app_ were 0.811–0.883, 0.773–0.858, and 0.761–0.892, respectively, reflecting good–excellent agreement (Table S5).

## 4. Discussion

This study explored whether qualitative and quantitative MRI features can differentiate between HER2-zero, -low, and -overexpression breast cancers, utilizing a simple and convenient imaging method. The findings indicate that qualitative and quantitative MRI features have distinct advantages depending on the lesion type. For mass lesions, combining qualitative features like intratumoral T2 hyperintensity and mass shape allowed for effective discrimination between HER2-low and HER2-zero breast cancers, with an AUC of 0.763. In contrast, for NME lesions, where qualitative features had limited utility, quantitative features (using the Combined_All_ model) distinguished HER2-low from HER2-overexpression cancers with an AUC of 0.802.

The development of HER2-targeted therapies has transformed treatment options for HER2-negative breast cancer, with HER2-low breast cancer patients now showing significant therapeutic benefits. Thus, accurately identifying HER2-low breast cancers from the traditional HER2 dichotomy is crucial for choosing appropriate treatment strategies. The features identified in this study can be detected through routine imaging, providing a more accessible and cost-effective approach to select patients who may benefit from targeted therapy. Furthermore, we discovered appropriate assessment methods for different lesion types, which have rarely been discussed in previous studies. Our findings contribute to the noninvasive preoperative assessment of low-HER2-expression breast cancer, and have significant implications for the precise diagnosis and targeted treatment of breast cancer.

A key finding in the qualitative analysis was that low-HER2-expression breast cancer had the highest proportion of intratumoral T2 hyperintensity. A possible explanation is that HER2-low breast cancers are more aggressive. Intratumoral T2 hyperintensity is typically considered a sign of necrosis, which results from tumor cell proliferation exceeding the vascular supply, leading to hypoxia in the tumor’s central region [24]. This feature is often associated with aggressive tumor behavior. The presence of T2 hyperintensity in HER2-low tumors could indicate their aggressive nature. Previous research has shown that HER2-low breast cancers are more aggressive, with the lowest pathological complete response rates to neoadjuvant therapy [7].

Another notable finding was the variation in lesion types across the HER2 expression groups. While studies have shown a link between HER2-positive breast cancer and NME, few have investigated low-HER2-expression breast cancer. Earlier studies suggested that the intraductal components of HER2-positive breast cancer are associated with the NME imaging phenotype [25,26]. Our results corroborate this, showing that the proportion of NME increases with HER2 expression levels. However, in our NME lesion analysis, the qualitative features on DCE-MRI had limited discriminatory value for HER2-low cancers. Conversely, for mass lesions, irregular shape emerged as an independent predictor of HER2-low breast cancer. This irregular shape may be linked to the higher positivity rates of ER and PR in HER2-low tumors in our study, consistent with findings by Szep et al., who noted that irregular masses are often associated with ER/PR-positive tumors [27].

While qualitative MRI features may not be highly informative for NME lesions, our study suggests that multi-b-value DKI histograms can effectively quantify HER2 expression levels in such lesions. This may indicate that our analysis of lesion distribution and internal enhancement patterns was insufficient to distinguish HER2-low breast cancer, and more refined analyses, such as those for quantitative features, are needed to better differentiate HER2-low breast cancer in NME lesions. Our analysis revealed that, in NME lesions, the mean, median, 5th percentile, ADC, and D_app_ values increased with HER2 expression levels, achieving statistical significance when comparing HER2-low with HER2-overexpressing tumors. The increase in HER2 expression may have two opposing effects on tissue: promoting cell proliferation, which restricts water diffusion, and inducing angiogenesis. This enhances diffusion by increasing vascular endothelial growth factor [28]. Our results suggest that, as HER2 levels rise, the angiogenic effects outweigh the cell proliferation effects, and this can be detected through ADC and D_app_ measurements.

The AUC analysis further demonstrated that K_app_ had the highest diagnostic efficacy among the diffusion parameters, and combining ADC, Dapp, and Kapp improved the discriminatory ability for HER2-low and HER2-overexpression breast cancers. This suggests that K_app_ may serve as a promising imaging biomarker for distinguishing HER2 expression levels. Multi-b-value DKI allows for the quantification of tissue microstructure complexity, reflected by higher K_app_ values [29]. In this study, several K_app_ histogram parameters were significantly elevated in HER2-low compared with HER2-overexpressing breast cancers, suggesting greater heterogeneity in HER2-low tumors. Since high heterogeneity is often associated with poor treatment response, future research could explore the role of DKI in assessing therapeutic outcomes.

This study has several limitations. First, it was a single-center retrospective study, and the sample size was relatively small. Although the data were derived from a consecutively collected group of patients and the fact that we controlled the selection criteria and the data collection process to ensure quality and reliability, selection bias may still exist. Further multicenter, large-sample studies are needed to validate the discriminatory performance of these MRI features. Second, the use of quantitative analysis led to the requirement of manual VOI drawing. While this method is simpler than radiomics analysis, it was more time-consuming than acquiring qualitative features. Future studies may benefit from utilizing automatic segmentation techniques to improve efficiency. Third, all images were acquired using the same instrument. While this allowed for consistent data acquisition, further research is necessary to assess whether the results hold across different imaging devices and parameters to improve the generalizability of the findings. Finally, the relatively short follow-up period in this study prevented us from evaluating the impact of MRI features on treatment response or long-term outcomes. However, this remains an important area for future research to explore the clinical relevance of MRI features in monitoring treatment efficacy and long-term patient prognosis.

## 5. Conclusions

In conclusion, our study demonstrates that qualitative and quantitative MRI features are valuable tools for noninvasively distinguishing HER2-low breast cancer, each showing unique strengths depending on the lesion type. For mass lesions, qualitative features such as irregular shape and intratumoral T2 hyperintensity are indicative of HER2-low expression. For NME lesions, quantitative features provide better discriminatory power, particularly when combined. These findings offer new insights into the noninvasive identification of HER2-low breast cancer in clinical practice, providing a more accessible and cost-effective approach to select patients who may benefit from targeted therapy.

## Figures and Tables

**Table 1:** Clinicopathological characteristics of patients according to HER2 expression levels.

Clinicopathological Characteristics	Total (n = 232); n (%)	HER2 Status Subgroup; n (%)			*p*-Value	*p*^1^-Value	*p*^2^-Value
		HER2-Zero (n = 60)	HER2-Low (n = 91)	HER2-Over (n = 81)		(Low vs. Zero)	(Low vs. Over)
Age (mean ± SD, years)	49.00 ±10.59	47.87 ± 10.40	48.41 ± 11.48	49.32 ± 9.74	0.709	NA	NA
Menopausal status					0.521	NA	NA
Pre-menopause	94 (40.5%)	23 (38.3%)	41 (45.1%)	30 (37.0%)			
Post-menopause	138 (59.5%)	37 (61.7%)	50 (54.9%)	51 (63.0%)			
ER status					<0.001	<0.001	<0.001
Positive	134 (57.8%)	30 (50.0%)	74 (81.3%)	30 (37.0%)			
Negative	98 (42.2%)	30 (50.0%)	17 (18.7%)	51 (63.0%)			
PR status					<0.001	<0.001	<0.001
Positive	104 (44.8%)	23 (38.3%)	63 (69.2%)	18 (22.2%)			
Negative	128 (55.2%)	37 (61.7%)	28 (30.8%)	63 (77.8%)			
HR status					<0.001	<0.001	<0.001
Positive	134 (57.8%)	30 (50.0%)	74 (81.3%)	30 (37.0%)			
Negative	98 (42.2%)	30 (50.0%)	17 (18.7%)	51 (63.0%)			
Ki-67 expression status (median, IQR)	40% (25%, 60%)						
Ki-67 expression status (cut-off 40%)					<0.001	<0.001	0.025
Low (<40%)	108 (46.6%)	16 (26.7%)	56 (61.5%)	36 (44.4%)			
High (≥40%)	124 (53.4%)	44 (73.3%)	35 (38.5%)	45 (55.6%)			
Size (median, IQR), mm)	40.00 (31.00, 53.75)	36.50 (26.25, 50.75)	40.00 (31.00, 52.00)	42.00 (31.00, 64.50)	0.352	NA	NA

Table 1 shows the clinicopathological characteristics of patients in HER2-zero, -low, and -overexpression groups. HER2, human epidermal receptor 2; SD, standard deviation; ER, estrogen receptor; PR, progesterone receptor; HR, hormone receptor; IQR, interquartile range; NA, not applicable. *p*: difference between the three groups. *p*^1^: HER2-low group compared with HER2-zero expression group; *p*^2^: HER2-low group compared with HER2-overexpression group.

**Table 2:** Qualitative MRI features according to HER2 expression levels.

MRI Features	HER2 Status Subgroup; n (%)			*p*-Value	*p*^1^-Value	*p*^2^-Value
	HER2-Zero	HER2-Low	HER2-Over		(Low vs. Zero)	(Low vs. Over)
All lesions (n = 232)						
Fibroglandular tissue				0.967	NA	NA
Almost entirely fat and scattered	13/60 (21.7%)	21/91 (23.1%)	19/81 (23.5%)			
Heterogeneous and extreme	47/60 (78.3%)	50/91 (54.9%)	62/81 (76.5%)			
Background parenchymal enhancement				0.206	NA	NA
Minimal or mild	47/60 (78.3%)	71/91 (78.0%)	71/81 (87.7%)			
Moderate or marked	13/60 (21.7%)	20/91 (22.0%)	10/81 (12.3%)			
Multifocal or Multicentric				0.550	NA	NA
Yes	20/60 (33.3%)	33/91 (36.3%)	34/81 (42.0%)			
No	40/60 (66.7%)	58/91 (63.7%)	47/81 (58.0%)			
Lesion type				0.017	0.094	0.145
Mass	46/60 (76.7%)	55/91 (60.4%)	40/81 (49.4%)			
NME	7/60 (11.7%)	14/91 (15.4%)	13/81 (16.0%)			
Mass with NME	7/60 (11.7%)	22/91 (24.2%)	28/81 (34.6%)			
Existence of NME				0.005	0.038	0.279
Yes	14/60 (23.3%)	36/91 (39.6%)	41/81 (50.6%)			
No	46/60 (76.7%)	55/91 (60.4%)	40/81 (49.4%)			
Intratumoral T2 hyperintensity				0.008	0.009	0.008
Present	28/60 (46.7%)	62/91 (68.1%)	39/81 (48.1%)			
Absent	32/60 (53.3%)	29/91 (31.9%)	42/81 (51.9%)			
Peritumoral edema				0.257	NA	NA
Present	35/60 (58.3%)	59/91 (64.8%)	58/81 (71.6%)			
Absent	25/60 (41.7%)	32/91 (35.2%)	23/81 (28.4%)			
Mass (n = 141)						
Shape				<0.001	<0.001	0.009
Oval or round	29/46 (63.0%)	11/55 (20.0%)	18/40 (45.0%)			
Irregular	17/46 (37.0%)	44/55 (80.0%)	22/40 (55.0%)			
Margin				<0.001	<0.001	0.659
Circumscribed	30/46 (65.2%)	16/55 (29.1%)	10/40 (25.0%)			
Not circumscribed	16/46 (34.8%)	39/55 (70.9%)	30/40 (75.0%)			
Internal enhancement				0.320	NA	NA
Homogeneous	1/46 (2.2%)	0/55 (0.0%)	0/40 (0.0%)			
Heterogeneous	30/46 (65.2%)	43/55 (78.2%)	30/40 (75.0%)			
Rim	14/46 (30.4%)	12/55 (21.8%)	8/40 (20.0%)			
Dark internal septations	1/46 (2.2%)	0/55 (0.0%)	2/40 (5.0%)			
NME (n = 91)						
Distribution				0.597	NA	NA
Linear	1/14 (7.1%)	0/36 (0.0%)	2/41 (4.9%)			
Segmental	6/14 (42.9%)	18/36 (50.0%)	20/41 (48.8%)			
Regional	4/14 (28.6%)	15/36 (41.7%)	14/41 (34.1%)			
Diffuse	3/14 (21.4%)	3/36 (8.3%)	5/41 (12.2%)			
Internal enhancement				0.733	NA	NA
Homogeneous	0/14 (0.0%)	0/36 (0.0%)	0/41 (0.0%)			
Heterogeneous	11/14 (78.6%)	24/36 (66.7%)	24/41 (58.5%)			
Clumped	3/14 (21.4%)	10/36 (27.8%)	15/41 (36.6%)			
Clustered ring	0/14 (0.0%)	2/36 (5.6%)	2/41 (4.9%)			

Table 2 shows the qualitative MRI features of patients in HER2-zero, -low, and -overexpression groups, including overall features of all lesions and characteristics specific to mass and NME lesions. NME, non-mass enhancement. NA, not applicable. *p*, difference between the three groups. *p*^1^, HER2-low group compared with HER2-zero group; *p*^2^, HER2-low group compared with HER2-overexpression group.

**Table 3:** Quantitative MRI features between HER2-low and other groups.

Quantitative MRI Parameters	HER2 Status Subgroup; n (%)			*p*-Value	*p*^1^-Value	*p*^2^-Value
	HER2-Zero (n = 60)	HER2-Low (n = 91)	HER2-Over (n = 81)		(Low vs. Zero)	(Low vs. Over)
ADC (10^−3^ mm^2^/s)						
ADC_Mean_	1.008 ± 0.181	1.062 ± 0.188	1.092 ± 0.181	0.001	1.000	0.005
ADC_Median_	0.987 ± 0.177	1.052 ± 0.192	1.077 ± 0.183	0.001	1.000	0.007
ADC5_%_	0.681 ± 0.150	0.708 ± 0.194	0.722 ± 0.160	<0.001	1.000	0.001
ADC_95%_	1.403 ± 0.283	1.450 ± 0.248	1.457 ± 0.264	0.313	NA	NA
ADC_Skewness_	0.536 ± 0.550	0.366 ± 0.506	0.386 ± 0.608	0.631	NA	NA
ADC_Kurtosis_	1.363 ± 1.771	1.393 ± 1.121	1.367 ± 1.599	0.249	NA	NA
ADC_Entropy_	2.986 ± 0.230	3.088 ± 0.163	2.99 ± 0.200	0.553	NA	NA
DKI-D_app_ (10^−3^ mm^2^/s)						
D_Mean_	1.204 ± 0.227	1.212 ± 0.199	1.305 ± 0.221	0.001	1.000	0.004
D_Median_	1.176 ± 0.225	1.191 ± 0.199	1.280 ± 0.225	0.002	1.000	0.007
D_5%_	0.849 ± 0.171	0.850 ± 0.172	0.952 ± 0.177	<0.001	1.000	0.012
D_95%_	1.659 ± 0.337	1.651 ± 0.285	1.734 ± 0.298	0.044	1.000	0.063
D_skewness_	0.662 ± 0.483	0.535 ± 0.487	0.506 ± 0.562	0.133	NA	NA
D_Kurtosis_	1.024 ± 1.584	0.966 ± 1.357	0.953 ± 1.622	0.111	NA	NA
D_Entropy_	2.669 ± 0.274	2.668 ± 0.242	2.678 ± 0.268	0.594	NA	NA
DKI-K_app_						
K_Mean_	0.978 ± 0.180	0.960 ± 0.168	0.897 ± 0.140	<0.001	0.700	0.001
K_Median_	0.947 ± 0.153	0.919 ± 0.154	0.874 ± 0.131	<0.001	0.696	0.002
K_5%_	0.600 ± 0.174	0.580 ± 0.176	0.593 ± 0.129	0.396	NA	NA
K_95%_	1.459 ± 0.453	1.458 ± 0.409	1.282 ± 0.328	<0.001	0.714	0.001
K_skewness_	1.306 ± 1.135	1.935 ± 3.165	1.472 ± 2.151	0.420	NA	NA
K_Kurtosis_	7.498 ± 9.989	26.481 ± 93.512	15.162 ± 59.754	0.589	NA	NA
K_Entropy_	1.143 ± 0.421	1.171 ± 0.383	0.920 ± 0.408	<0.001	1.000	<0.001

Table 3 shows the quantitative MRI features of patients in HER2-zero, -low, -overexpression groups, including the mean, median, 5th percentile, 95th percentile, skewness, kurtosis, and entropy, which are displayed in subscript notation, for the ADC, D_app_, and K_app_. ADC, apparent diffusion coefficient; DKI, diffusion kurtosis imaging. *p*, difference between the three groups. *p*^1^: HER2-low group compared with HER2-zero expression group; *p*^2^: HER2-low group compared with HER2-overexpression group. NA, not applicable.

**Table 4:** ROC analysis of quantitative MRI features differentiating HER2-low and HER2-overexpression breast cancer in NME-related lesions.

MRI Features	AUC	95% CI	Sensitivity%	Specificity%	Cut-Off Value
ADC_Mean_	0.668	0.543–0.794	95.12	38.89	0.970
ADC_Median_	0.666	0.541–0.791	95.12	38.89	0.957
ADC_5%_	0.693	0.571–0.815	90.24	55.56	0.702
Combined_ADC_	0.694	0.573–0.815	50.00	90.24	0.454
D_Mean_	0.671	0.545–0.796	90.24	44.44	1.199
D_Median_	0.667	0.542–0.792	78.05	58.33	1.244
D_5%_	0.683	0.558–0.808	92.68	50.00	0.839
Combined_D_	0.681	0.556–0.806	50.00	92.68	0.429
K_Mean_	0.680	0.555–0.804	78.05	58.33	0.925
K_Median_	0.667	0.541–0.792	75.61	61.11	0.883
K_95%_	0.729	0.615–0.843	87.80	52.78	1.359
K_Entropy_	0.776	0.666–0.887	65.85	91.67	0.725
Combined_K_	0.786	0.678–0.894	91.67	65.85	0.666
Combined_all_	0.802	0.701–0.903	86.11	70.73	0.621

Table 4 shows the ROC analysis of quantitative MRI features for distinguishing between HER2-low and -overexpression breast cancer, including the AUC, 95% CI, sensitivity, specificity, and cut-off values for both single parameters and combined models. ROC, receiver operator characteristic curve; AUC, area under the curve; CI, confidence interval. Combined_ADC_, Combined_D_, and Combined_K_ refer to models combining significant indicators from the ADC, D_app_, and K_app_ histograms, respectively. Combined_All_ is the comprehensive model that combines all significant indicators from the three histograms.

## Data Availability

The datasets presented in this article are not readily available because the data are part of an ongoing study.

## Supplementary Materials

The following supporting information can be downloaded at: https://www.mdpi.com/article/10.3390/tomography11030031/s1, Table S1: Sequence parameters of MRI scanning; Table S2: Multivariate logistic regression analysis in mass lesions between HER2-low and other groups; Table S3: Quantitative MRI features in NME-related lesions between HER2-low and other groups; Table S4: Quantitative MRI features in mass lesions between HER2-low and other groups; Table S5: Interobserver Agreement Analysis.
