# Supplementary material for: Distinguishing Low Expression Levels of Human Epidermal Growth Factor Receptor 2 in Breast Cancer: Insights from Qualitative and Quantitative Magnetic Resonance Imaging Analysis
Source: Tomography. 2025 Mar 10;11(3):31. doi: 10.3390/tomography11030031 (PMC11945706; doi:10.3390/tomography11030031)
Supplement: Supplementary file 1 [file tomography-11-00031-s001.zip › tomography-3441443-supplementary.pdf]

Table S1 Sequence parameters of MRI scanning

Parameters	T1WI	T2WI	DCE-MRI	DKI
Repetition time (TR)/ms	5.4	3570	4.5	6700
Echo time (TE)/ms	2.46	69	1.6	65
Field of view (FOV)/mm^2	350×350	350×350	340×340	318×239
Slice thickness/mm	2.2	5	1.5	5
Gap/mm	0.4	0.6	0	0
Slice	80	34	80	28
Acquisition time/s	50	79	360	491
b-values (s/mm^2)	/	/	/	0, 800, 1400, 2000

Table S1 shows the scanning parameters of the MRI sequences used in the study. T1WI, T1-weighted imaging; T2WI, T2-weighted imaging; DCE, dynamic contrast enhancement; DKI, diffusion kurtosis imaging.

Table S2 Multivariate logistic regression analysis in mass lesions between HER2-low and other groups

**Variables**	**HER2-low vs. HER2-zero expression**		**HER2-low vs. HER2-over expression**	
	OR (95% CI)	*P*-value	OR (95% CI)	*P*-value
Mass-shape				
Oval or round (Reference)				
Irregular	3.91 (1.38, 11.05)	0.010	2.23 (1.29, 8.10)	0.012
Intratumoral T2 hyperintensity				
Absent (Reference)				
Present	0.50 (0.16, 1.20)	0.229	0.77 (0.21, 1.27)	0.511

Table S2 shows the multivariate logistic regression results of qualitative MRI features in mass lesions for distinguishing HER2-low breast cancer and HER2-zero or HER2-overexpression breast cancer. HER2, human epidermal receptor 2; OR, odds ratio; CI, confidence interval.

Table S3 Quantitative MRI features in NME-related lesions between HER2-low and other groups

Quantitative MRI parameters	HER2 status subgroup; n (%)			*P*-value	*P*[1]-value	*P*[2]-value
	HER2-zero (n=14)	HER2-low (n=36)	HER2-over （n=41）		(low vs. zero)	(low vs. over)
ADC						
ADC_{Mean}	1.020 ± 0.159	1.062 ± 0.188	1.152 ± 0.135	**0.007**	1.000	**0.030**
ADC_{Median}	1.004 ± 0.165	1.052 ± 0.192	1.146 ± 0.140	**0.006**	1.000	**0.034**
$ADC5_{\%}$	0.654 ± 0.167	0.708 ± 0.194	0.825 ± 0.140	**0.001**	1.000	**0.008**
$ADC_{95\%}$	1.438 ± 0.200	1.450 ± 0.248	1.504 ± 0.180	0.313	NA	NA
$ADC_{Skewness}$	0.329 ± 0.438	0.366 ± 0.506	0.240 ± 0.567	0.631	NA	NA
$ADC_{Kurtosis}$	0.952 ± 0.973	1.393 ± 1.121	1.202 ± 1.289	0.249	NA	NA
$ADC_{Entropy}$	3.136 ± 0.123	3.088 ± 0.163	3.058 ± 0.191	0.553	NA	NA
DKI-D_{app}						
D_{Mean}	1.236 ± 0.193	1.274 ± 0.231	1.386 ± 0.164	**0.008**	1.000	**0.025**
D_{Median}	1.213 ± 0.202	1.257 ± 0.234	1.371 ± 0.171	**0.008**	1.000	**0.028**
$D_{5\%}$	0.833 ± 0.175	0.892 ± 0.217	1.014 ± 0.154	**0.001**	1.000	**0.012**
$D_{95\%}$	1.714 ± 0.228	1.720 ± 0.293	1.799 ± 0.208	0.284	NA	NA
$D_{skewness}$	0.478 ± 0.403	0.471 ± 0.505	0.313 ± 0.493	0.133	NA	NA
$D_{Kurtosis}$	0.540 ± 0.906	0.959 ± 0.973	0.700 ± 1.281	0.111	NA	NA
$D_{Entropy}$	2.801 ± 0.175	2.736 ± 0.229	2.729 ± 0.289	0.594	NA	NA
DKI-K_{app}						
K_{Mean}	1.010 ± 0.158	0.960 ± 0.168	0.870 ± 0.129	**0.001**	0.700	**0.015**
K_{Median}	0.956 ± 0.115	0.919 ± 0.154	0.847 ± 0.114	**0.002**	0.696	**0.033**
$K_{5\%}$	0.631 ± 0.141	0.580 ± 0.176	0.615 ± 0.100	0.396	NA	NA
$K_{95\%}$	1.553 ± 0.447	1.458 ± 0.409	1.201 ± 0.256	**<0.001**	0.714	**0.002**
$K_{skewness}$	1.775 ± 0.985	1.935 ± 3.165	1.476 ± 1.860	0.420	NA	NA

$K_{Kurtosis}$	9.410 ± 8.219	26.481 ± 93.512	12.668 ± 34.974	0.589	NA	NA
$K_{Entropy}$	1.209 ± 0.313	1.171 ± 0.383	0.798 ± 0.359	**<0.001**	1.000	**<0.001**

Table S3 shows the quantitative MRI features of patients with NME-related lesions in HER2-zero, -low, -over expression groups, including the mean, median, 5_{th} percentile, 95_{th} percentile, skewness, kurtosis, and entropy, which are displayed in subscript notation, for the ADC, D_{app} and K_{app} histograms. NME, nonmass enhancement; ADC, apparent diffusion coefficient; DKI, diffusion kurtosis imaging. *P*, difference between the three groups. P^1: HER2-low group compared with HER2-zero expression group; P^2: HER2-low group compared with HER2 overexpression group. NA, not applicable;

Table S4 Quantitative MRI features in mass lesions between HER2-low and other groups

Quantitative MRI parameters	HER2 status subgroup; n (%)			*P*-value
	HER2-zero (n=46)	HER2-low (n=55)	HER2-over （n=40）	
ADC				
ADC_{Mean}	1.004 ± 0.189	0.991 ± 0.139	0.991 ± 0.139	0.552
ADC_{Median}	1.983 ± 0.182	0.974 ± 0.132	0.973 ± 0.132	0.648
$ADC5_{\%}$	0.690 ± 0.147	0.668 ± 0.127	0.668 ± 0.127	0.192
$ADC_{95\%}$	1.393 ± 0.305	1.364 ± 0.244	1.363 ± 0.244	0.756
$ADC_{Skewness}$	0.599 ± 0.569	0.446 ± 0.532	0.446 ± 0.532	0.563
$ADC_{Kurtosis}$	1.489 ± 1.941	1.287 ± 1.536	1.287 ± 1.536	0.810
$ADC_{Entropy}$	2.939 ± 0.236	2.963 ± 0.196	2.963 ± 0.197	0.750
DKI-D_{app}				
D_{Mean}	1.195 ± 0.237	1.173 ± 0.165	1.172 ± 0.165	0.523
D_{Median}	1.165 ± 0.232	1.148 ± 0.161	1.148 ± 0.161	0.664
$D_{5\%}$	0.854 ± 0.172	0.823 ± 0.132	0.824 ± 0.132	0.094
$D_{95\%}$	1.642 ± 0.364	1.606 ± 0.274	1.605 ± 0.274	0.624
$D_{skewness}$	0.717 ± 0.496	0.576 ± 0.474	0.577 ± 0.474	0.309
$D_{Kurtosis}$	1.172 ± 1.721	0.971 ± 1.567	0.971 ± 1.567	0.756
$D_{Entropy}$	2.629 ± 0.287	2.623 ± 0.242	2.623 ± 0.242	0.827
DKI-K_{app}				
K_{Mean}	0.969 ± 0.187	0.973 ± 0.134	0.973 ± 0.134	0.190
K_{Median}	0.944 ± 0.164	0.947 ± 0.118	0.947 ± 0.118	0.194
$K_{5\%}$	0.591 ± 0.183	0.605 ± 0.154	0.605 ± 0.154	0.444
$K_{95\%}$	1.432 ± 0.457	1.426 ± 0.286	1.426 ± 0.286	0.307
$K_{skewness}$	1.164 ± 1.149	1.242 ± 1.185	1.242 ± 1.185	0.919

$K_{Kurtosis}$	6.916 ± 10.479	7.28 ± 10.299	7.281 ± 10.299	0.933
$K_{Entropy}$	1.123 ± 0.451	1.175 ± 0.369	1.175 ± 0.369	0.217

Table S4 shows the quantitative MRI features of patients with mass lesions in HER2-zero, -low, -over expression groups, including the mean, median, 5_{th} percentile, 95_{th} percentile, skewness, kurtosis, and entropy, which are displayed in subscript notation, for the ADC, D_{app} and K_{app} histograms. ADC, apparent diffusion coefficient; DKI, diffusion kurtosis imaging. *P*, difference between the three groups. Due to the lack of significant differences between the three groups, no further comparisons were made between HER2-low and the other groups.

Table S5 Interobserver Agreement Analysis

MRI features	κ /ICC (95% CI)	MRI features	ICC (95% CI)
Fibroglandular tissue	0.895	$ADC_{Kurtosis}$	0.883 (0.801, 0.932)
BPE	0.916	$ADC_{Entropy}$	0.822 (0.583, 0.914)
Multifocal or Multicentric	0.945	D_{Mean}	0.831 (0.695, 0.905)
Lesion type	0.922	D_{Median}	0.852 (0.742, 0.915)
Intratumoral T2 hyperintensity	0.887	$D_{5\%}$	0.822 (0.685, 0.900)
Peritumoral edema	0.870	$D_{95\%}$	0.841 (0.643, 0.921)
Mass-Shape	0.922	$D_{skewness}$	0.858 (0.763, 0.917)
Mass-Margin	0.915	$D_{Kurtosis}$	0.808 (0.677, 0.888)
Mass-Internal enhancement	0.957	$D_{Entropy}$	0.773 (0.517, 0.885)
NME-Distribution	0.820	K_{Mean}	0.892 (0.802, 0.940)
NME-Internal enhancement	0.834	K_{Median}	0.828 (0.700, 0.902)
ADC_{Mean}	0.826 (0.710, 0.898)	$K_{5\%}$	0.799 (0.672, 0.881)
ADC_{Median}	0.862 (0.769, 0.919)	$K_{95\%}$	0.825 (0.697, 0.900)
$ADC5_{\%}$	0.811 (0.683, 0.889)	$K_{skewness}$	0.779 (0.588, 0.879)
$ADC_{95\%}$	0.853 (0.741, 0.917)	$K_{Kurtosis}$	0.761 (0.613, 0.857)
$ADC_{Skewness}$	0.859 (0.763, 0.917)	$K_{Entropy}$	0.795 (0.629, 0.886)

Table S5 shows the interobserver agreement analysis, with kappa coefficients for qualitative features and ICC for quantitative features. κ , Kappa Coefficient. ICC, Intraclass Correlation Coefficient. CI, confidence interval.
